# An unusual association between Burkitt’s lymphoma and a Carcinoid tumor in a Syrian boy: a rare case report

**DOI:** 10.1097/MS9.0000000000000663

**Published:** 2023-04-11

**Authors:** Haidara Kherbek, Marah Hinawi, Khedr Layka, Yana Hleibieh, Khawla Jaber, Roukaya Jaber, Zuheir Alshehabi

**Affiliations:** a Faculty of Medicine; b Department of Pathology, Cancer Research Center, Tishreen University, Latakia, Syria

**Keywords:** Burkitt’s lymphoma, case report, carcinoid tumor, immunohistochemistry, neuroendocrine tumor

## Abstract

Burkitt’s lymphoma (BL), an aggressive kind of non-Hodgkin’s B-cell lymphoma. On the other hand, appendiceal carcinoid tumors are uncommon neuroendocrine neoplasms.

**Case presentation:**

The authors report a case of a 15-year-old Syrian adolescent who was admitted to our hospital due to a persistent, severe generalized abdomen pain accompanied by nausea, vomiting, loss of appetite, and inability to pass stool or gas. An abdominal radiograph revealed dilated intestinal loops with air-fluid levels. The patient underwent emergency surgery through which a retroperitoneal mass was removed as well as part of the ileum and the appendix. The final diagnosis was consistent with intestinal BL associated with an appendiceal carcinoid tumor.

**Discussion:**

The correlation between gastrointestinal carcinoids and other types of tumors was frequently reported. However, there have been few reports of carcinoid tumors being associated with lymphoreticular system cancers. BLs were classified into three variants: endemic, sporadic, and acquired immunodeficiency-associated BL while appendiceal neuroendocrine tumors were classified as the following: well-differentiated neuroendocrine tumors with benign or uncertain malignant potential; well-differentiated neuroendocrine carcinoma with low malignant potential; and mixed exocrine-neuroendocrine carcinoma.

**Conclusion:**

Our article demonstrates an unusual association between BL and an appendiceal carcinoid tumor that highlights the significant role of histological and immunohistochemical staining in confirming the diagnosis, as well as the role of surgery in treating the complications of intestinal BLs.

## Introduction

Highlights

- Burkitt’s lymphoma is an aggressive kind of non-Hodgkin’s B-cell lymphoma.
- Appendiceal carcinoid tumors are uncommon benign neuroendocrine neoplasms.
- There have been few reports of carcinoid tumors being associated with lymphoreticular system cancers.
- Chemotherapy is the mainstay of Burkitt’s lymphoma.
- Surgery is the main treatment of appendiceal carcinoid tumor.

Burkitt’s lymphoma (BL), an aggressive kind of non-Hodgkin’s B-cell lymphoma, typically identified in children and young adults. It has three clinical-epidemiological variants: endemic, sporadic, and acquired immunodeficiency-associated BL^1^. On the other hand, appendiceal carcinoid tumors are uncommon neuroendocrine neoplasms, which are usually benign, though some lesions may be potentially malignant and can therefore metastasize^2^. They are frequently discovered by chance when having an appendectomy or other abdominal surgery^2^ gastrointestinal (GI) tract carcinoid tumors are frequently associated with different kinds of tumors, the majority of these tumors are adenocarcinomas of the GI tract; however, other types and locations of tumors have also been described. The accompanying neoplasm and the carcinoid may develop together or separately^3^. Herein, we report a case of a 15-year-old male who was admitted to our hospital and diagnosed with enteric BL and carcinoid of the appendix.

## Case presentation

We report a case of a 15-year-old Syrian adolescent who was admitted to our hospital due to a persistent, severe generalized abdomen pain accompanied by nausea, vomiting, loss of appetite, and inability to pass stool or gas. Family history was unremarkable as well as surgical, drug, and allergic histories. Physical examination revealed a soft, tender abdomen without guarding. Lab findings were significant for leukocytosis (14×10^9^ /l) and elevated levels of C-reactive protein (value 32 mg/dl), otherwise normal.

**Figure 1:**
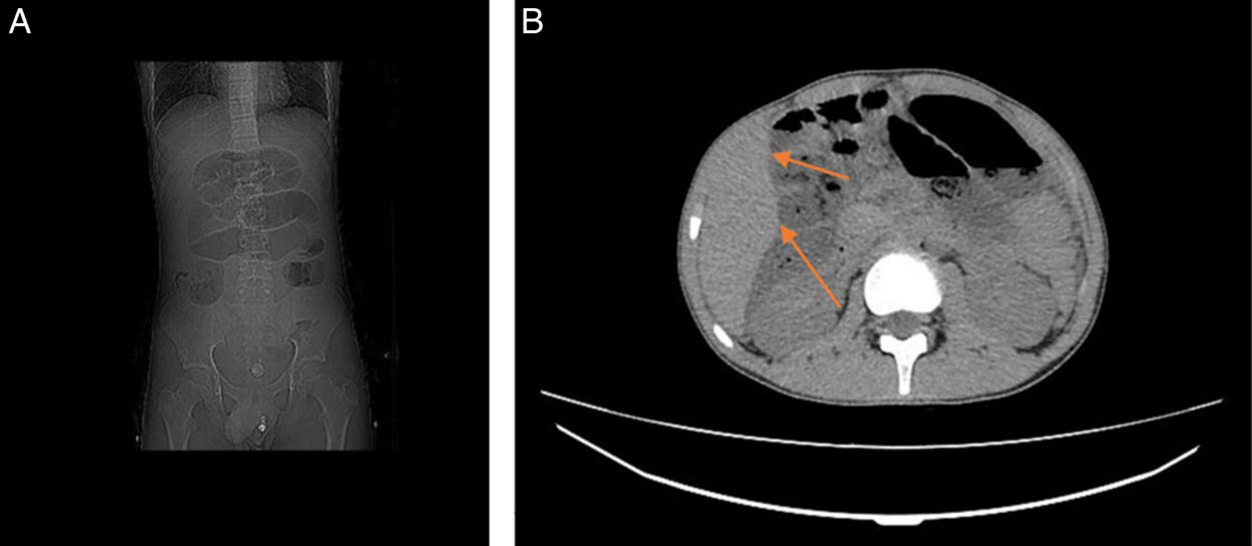
(A) Abdominal radiograph showing multiple fluid-gas levels, (B) Abdominal computed-tomography showing segmental-wall thickening (arrows) with fluid-gas levels.

**Figure 2:**
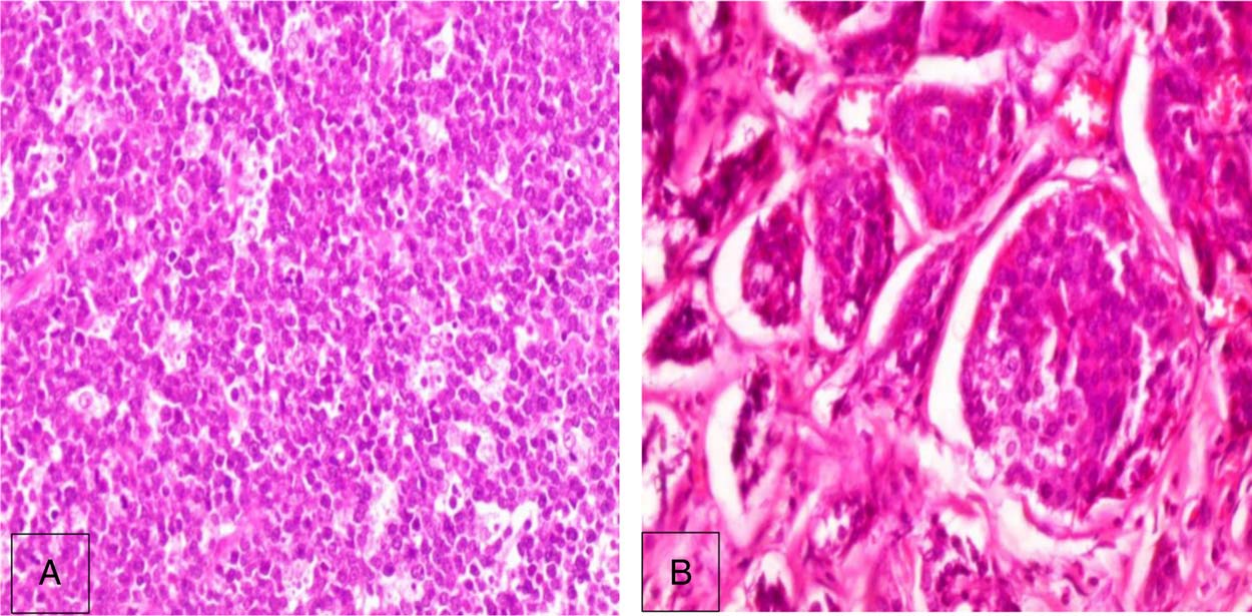
(A) Sheets of monotonous intermediate-sized cells with starry sky appearance consistent with Burkitt's lymphoma (H&E×200), (B) uniform, polygonal-shaped tumor cells with round to oval nuclei and eosinophilic cytoplasm (H&E×200).

**Figure 3:**
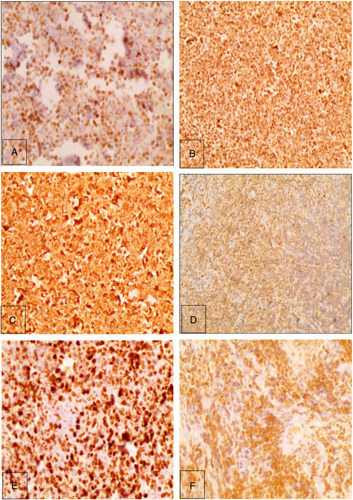
Immunohistochemistry staining showing positivity of: (A) BCL-6, (B) BCL-2, (C) CD20,(D) CD10,(E) KI67 of 100%, (F) Chromogranin-A.

Abdominal radiograph revealed dilated intestinal loops with air-fluid levels (Fig. 1A). An abdominal and pelvic computed-tomography scan demonstrated dilated intestinal loops with segmental-wall thickening (Fig. 1B). The patient underwent emergency surgery, through which a retroperitoneal mass was removed as well as part of the ileum and the appendix. Using general anesthesia, the surgeon performed a supraumbilical and infraumbilical median incisions to reach the abdominal cavity, then he performed a retroperitoneal dissection and eradicated the retroperitoneal mass that had reached the ileum. An ileectomy with a subsequent ileo-ileostomy were also done. Then the surgeon put a drainage in the Douglas diverticulum and removed the appendix. Prior to surgery, some precautionary measures were taken to reduce the probability of infection, such as using an alcoholic antiseptic to decontaminate the skin and administering prophylactic antibiotics. Seven days later, the patient was discharged with no postsurgical complications. Gross examination showed an appendix of 9.5 cm long, attached to a nodular mass measuring 1.5 cm, and an enteric segment of 85 cm with a rubbery gray lobed mass. The microscopic exam of the retroperitoneal mass and the enteric segment showed sheets of monotonous intermediate-sized, high-grade cells with starry sky appearance (Fig. 2A) consistent with BL, which was further confirmed by the positivity of the following immune stains: BCL-6 (Fig. 3A), BCL-2 (Fig. 3B), CD20 (Fig. 3C), CD10 (Fig. 3D), and KI67 of 100% (Fig. 3E), and the negativity of CD3 and CD5. On the other hand, the histological examination of the appendix and the attached nodule revealed uniform, polygonal-shaped, low-grade tumor cells with round to oval nuclei and eosinophilic cytoplasm (Fig. 2B) consistent with carcinoid tumor, which was further confirmed by the positivity of the chromogranin-A (CgA) (Fig. 3F). Thus, the final diagnosis of stage II BL and localized T2N0M0 appendiceal carcinoid tumor was made. The prognosis is good. The treatment plan included alternating sessions of cyclophosphamide, vincristine, prednisone, adriamycn, and methotrexate, then courses of cytarabine and methotrexate. The patient will be followed at periodic intervals of 3 months for the first 2 years. Then at intervals of 6 months for the next 3 years.

## Discussion

Primary GI non-Hodgkin lymphoma is considered a rare entity, affecting 10–15% of all NHL patients and accounting for only 1–4% GI neoplasms^4^. Based on its geographic distribution, BLs were classified into the following subtypes: endemic BL, which is primarily located in Africa and affects the facial skeleton in children aged two-to-nine. Sporadic BL, which is the form found outside Africa, affecting the abdominal viscera and can be detected at any age. However, a third subtype was proposed and included BL associated with HIV infection^5^. Histologically, BL must be distinguished from other types of lymphoma especially diffuse large B-cell lymphoma (DLBCL), as well as precursor B-lymphoblastic lymphoma, precursor T-lymphoblastic lymphoma, a blastoid variant of mantle cell lymphoma, and florid follicular hyperplasia^6^. This can be achieved based on the histological and immunohistochemical features of each lesion^6^. According to literature, BLs express monotypic small IgM (sIg), pan-B-cell antigens, including CD19, CD20, CD22, and CD79a, and coexpresses CD10, Bcl-6, CD43, and p53, but not CD5, CD23, Bcl-2, CD138, or terminal deoxynucleotidyl transferase^7^. On the other hand, DLBCLs are histologically characterized by the presence of neoplastic cells with large oval, irregular/lobated nuclei, and scant cytoplasm in addition to the expression of CD10, CD20, BCL-6, BCL-2, monotypic sIg^6^. In 2011, a study performed by Pervez, Shahid *et al*.^8^ stated that the positivity of BCL-2 is not a reliable marker to differentiate DLBCLs from BLs as a strong BCL-2 expression was found in up to 10% of studied cases. Precursor B and T-lymphoblastic lymphoma have similar morphologic features. However, precursor B- lymphoblastic lymphoma is characterized by positive CD19, CD10, CD20 immune stains, but not sIg whereas the T-type characterized by positive CD1a, CD3, CD4/8, and CD7 immune stains^6^. Mantle cell lymphoma express CD10, CD20, BCL-2, polytypic Ig, cyclin D1, and have a very high proliferation rate (~100%)^6^. Finally, florid follicular hyperplasia is characterized by expression of CD10, CD20, BCL-6, polytypic Ig but not BCL-2 and it has a very high proliferation rate (~100%)^6^. Most cases of BL were among children aged five-to-fifteen, with a higher incidence among males^9^. BL primarily affects the abdomen, including the mesenteric and retroperitoneal lymph nodes. However, it is mainly found in the ileo-cecal region^10^, appearing either as a segmental-wall thickening or a mass communicated to the lumen, which may be complicated by bowel intussusception and subsequently obstruction^11^. In such cases, ultrasonography may reveal the ‘target’ sign whereas computed-tomography may show a telescoping bowel^10^. The main treatment of BL is chemotherapy with no role to radiotherapy. However, surgery is not performed until serious complications has happened such as bowel obstruction^12^. On the other hand, appendiceal neuroendocrine tumors (NETs) comprises more than half of all primary tumors of the appendix and despite that, they are still considered a rare entity^13^.

Appendiceal NETs were classified as the following: well-differentiated NETs with benign or uncertain malignant potential; well-differentiated neuroendocrine carcinoma with low malignant potential; and mixed exocrine-neuroendocrine carcinoma^13^. The differential diagnosis for appendiceal carcinoid includes: appendiceal adenocarcinoma, signet ring cell tumor, goblet cell carcinoma, and appendiceal mucinous-benign tumors^14^. According to the literature , most cases of appendiceal carcinoid tumors were among adults in their third or fourth decades, as children tend to have lower prevalence rates ranging from 0.2–0.5%^15^.

The majority of individuals with appendiceal carcinoids have tumors smaller than 1 cm, making appendectomy the only necessary treatment^2^. The correlation between GI carcinoids and other types of tumors was frequently reported, such as adenocarcinoma, squamous cell carcinoma, gliomas, mesotheliomas, urothelial carcinomas, hepatomas, and leiomyosarcomas. However, there have been few reports of carcinoid tumors being associated with lymphoreticular system cancers^3^. According to the literature, there are eight case reports of carcinoids with associated lymphpreticular system malignancies; two of these cases involved non-Hodgkin lymphomas of the GI tract, while the topographical location was not mentioned in four cases^3^. Some studies stated that a relationship between NETs and lymphoreticular tumors does exist, as they both share some signaling molecules and receptors, indicating that neuroendocrine hormones may have an impact on the proliferation and mitogenesis of lymphoid cells and diseases might develop as a result of changes in this pathway^16^.

## Conclusion

Our article demonstrates an unusual association between BL and an appendiceal carcinoid tumor that highlights the significant role of histological and immunohistochemical staining in confirming the diagnosis, as well as the role of surgery in treating the complications of intestinal BLs. Therefore, in future cases, at least precise histological and immunohistochemical exams should be performed as they are the key to make such diagnoses clear.

## Methods

Our work was reported according to the SCARE 2020 criteria^17^.

## Ethical approval

Not applicable.

## Informed consent

Written informed consent was obtained from the patient for publication of this case report and accompanying images. A copy of the written consent is available for review by the Editor-in-Chief of this journal on request.

## Sources of funding

Self-funding.

## Author contribution

H.K. is the mentor and drafted the manuscript. M.H., K.L., Y.H., R.J., and K.J. collected the data and participated in drafting the manuscript. Z.A. is a professor of pathology and critically reviewed the manuscript.

## Conflicts of interest disclosure

The authors declares that they have no conflicts of interest.

## Research registration unique identifying number (UIN)

1. Name of the registry: Not applicable.
2. Unique Identifying number or registration ID: Not applicable.
3. Hyperlink to your specific registration (must be publicly accessible and will be checked): Not applicable.

## Provenance and peer review

Not commissioned, externally peer-reviewed.
